# Population aging and the spatial equity of prehospital emergency medical resources: evidence from Shanghai's five new towns

**DOI:** 10.3389/fpubh.2026.1948408

**Published:** 2026-10-12

**Authors:** Xiaojian Zheng, Menglan Zhu, Xiaojun Tang, Zhibin Chen, Jianqiang He, Zhifeng Zhang

**Affiliations:** 1 Shanghai Medical Emergency Center, Shanghai, China; 2 Department of Ophthalmology and Otolaryngology, Unit 32265 of the People's Liberation Army, Guangzhou, China; 3 Jincheng Emergency Medical Rescue Center, Jincheng, Shanxi, China; 4 Shanghai Fengxian District Medical Emergency Center, Shanghai, China

**Keywords:** dual-track equity framework, emergency response time, healthcare resource allocation, population aging, prehospital emergency care, spatial equity

## Abstract

**Background:**

China is undergoing rapid urbanization and profound population aging, presenting the prehospital emergency medical system with the dual challenges of inadequate capacity and spatial mismatch. Shanghai's five new towns are strategic vehicles for the spatial restructuring of a megacity; however, evidence is still lacking as to whether their prehospital emergency systems can meet the aging-driven increase in demand.

**Objective:**

To evaluate the spatial equity of emergency medical resource allocation in the five new towns and to analyze its associations with population aging and future demand.

**Methods:**

Based on 2024 data, the Gini coefficient and Theil index were used to evaluate equity; population projections were performed using the cohort-component method in PADIS-INT (population analysis software); resource requirements were estimated in stages according to policy standards; and coverage gaps were identified through isochrone analysis in QGIS (open-source geographic information system).

**Results:**

The population-based Gini coefficients across the five districts ranged from 0.03 (substations) to 0.10 (physicians), indicating relatively equitable per capita allocation. However, after including all 16 districts of the municipality, the area-based Gini coefficient for substations reached 0.61, reflecting a gradient in substation density between the urban core and the suburbs. Under the medium scenario, the population aged 65 years and older in Jiading increases from 13.18% to 19.15% (2035), and the demand for R1 standard ambulances in Jiading New City is projected to rise from 24 vehicles in 2025 to 29 vehicles in 2035. In Fengxian, patients aged 60 years and above accounted for 48.49% of emergency calls. Highly aged subdistricts spatially overlapped with 8 min coverage blind spots. Notably, stratified emergency response time (ERT) analysis showed that in two of the three districts, the differences in ERT between cardiac arrest and non-emergency patients were not statistically significant (*p* > 0.05), suggesting limited implementation of dispatch prioritization.

**Conclusion:**

The five new towns exhibit a dual-track pattern of equity in population terms but inequity in spatial terms. Population aging exacerbates inequity through demand expansion, changes in the disease spectrum, and spatial overlap.

## 1 Introduction

### 1.1 Emergency care challenges under the compounded effects of rapid urbanization and population aging

Prehospital emergency care is the first line of defense in the public health emergency response. China has established an emergency medical services network covering more than 300 prefecture-level cities. Shanghai's emergency medical system ranks among the top nationwide in both scale and total resources.

Meanwhile, China has the world's largest and most rapidly aging population (1, 2). Older adults use emergency services more frequently and present unique physiological challenges: age-related declines in physiological reserve, multimorbidity, and polypharmacy alter patterns of response to injury and often obscure early signs of clinical deterioration (3). This reduces the sensitivity of standard prehospital triage tools for older populations, resulting in systematic under-triage and delaying opportunities for definitive treatment (4). In addition, the social determinants of health—including socioeconomic status, residential environment, and social support networks—significantly influence older adults' injury risk and recovery trajectories (5, 6). Megacities are transitioning from monocentric to polycentric structures; determining whether emergency care resource allocation keeps pace with the growth in aging-related demand at these new urban nodes is a critical policy question. The systematic review by Günay Tuzcu et al. (7) further corroborated that the intensity of emergency care demand in older populations is significantly higher than in younger populations and accelerates with advancing age. The World Health Organization, in its Baseline report for the Decade of Healthy Ageing (8), emphasized that population aging poses a systemic challenge to global health systems, and that emergency care systems, as the first line of defense in health emergencies, should be prioritized in the response.

### 1.2 The five new towns of Shanghai: research context and policy background

Shanghai's new-town programme has evolved in two stages. The “One City, Nine Towns” initiative (2001) designated nine experimental towns—Anting, Luodian, Zhoupu, Fengcheng, Gaoqiao, Pujiang, Zhujiajiao, Fengjing, and Chenjiazhen—as low-density development nodes on the urban fringe. In the 14th Five-Year Plan (2021), the strategy was upgraded and concentrated on five new towns: Jiading, Qingpu, Songjiang, Fengxian, and Nanhui. Unlike the earlier pilot towns, these five are designated as independent, comprehensive urban nodes, each with a delimited planning boundary, dedicated infrastructure investment, and quantitative 2035 population targets. We focus on these five towns for three reasons: (i) they are the current policy priority and the explicit object of EMS planning standards; (ii) their delimited planning boundaries permit core-area-specific spatial analysis; and (iii) the operational data used here (station inventories and call records) are available for their host districts.

The development of new towns faces threefold challenges: a surge in population accompanied by an expanding aging cohort; expansion of service radii with a concomitant decline in site density; and a dual insufficiency in both the quantity and quality of resources.

Accordingly, policy expectations for the emergency medical services system have become increasingly refined (9–14). However, existing evidence has primarily focused on station siting optimization (15, 16) or single-dimensional equity assessment (17, 18). Few studies have integrated population aging projections, multidimensional spatial equity measurement, and estimation of resource requirements under policy standards into a single analytical framework (19, 20), particularly for planned new towns as a distinctive urban form. The study by Shi et al. (21) on the accessibility of healthcare services in Chinese new towns provides an important comparative perspective for this study, and its findings corroborate the spatial equity results presented herein.

However, whether these goals can be achieved hinges on an unanswered question: Is the current allocation of emergency medical resources sufficient to meet demand changes driven by population aging?

### 1.3 Core research questions and contributions

The core question is: What is the association between population aging and the spatial equity of emergency medical resources in the five New Towns?

We operationalize this question through a dual-track equity framework that differentiates population-dimension equity (per capita resource allocation) and spatial-dimension equity (geographic distribution and coverage area of stations).

This study contributes to the existing literature in three respects. First, we propose a dual-track equity framework that distinguishes equity along population and spatial dimensions, revealing patterns that single-dimension measures may overlook. Second, we integrate population aging projections with spatial coverage analysis to identify future risk areas where aging hotspots overlap with current service gaps. Third, we provide evidence on triage priorities at the scheduling level, offering actionable insights for operational improvements beyond facility planning.

## 2 Methods

### 2.1 Study design and scope

This study employed a descriptive, policy-oriented cross-sectional analysis combined with prospective population simulation, covering the five new towns of Shanghai (Jiading, Qingpu, Songjiang, Fengxian, Nanhui). Each new town is a planned core area within its host administrative district; Nanhui New Town lies within Pudong New Area (the former Nanhui District was merged into Pudong in 2009). Here, “new town core area” denotes the group of towns and subdistricts within a new town's planning boundary, which serve as the representative units for new-town-specific data extraction. Unless otherwise specified, analyses are reported at two scales: the district scale (district totals; Sections 3.1.1, 3.2, and 3.3) and the new town core area scale (Sections 3.1.2–3.1.3 and 3.4).

### 2.2 Data sources

Population and planning data were derived from the 2020 Seventh National Population Census age and sex data, the 2035 population target, and a demographic study based on death registration in Jiading District, Shanghai (22). Because full age- and sex-specific population structure is published only by the decennial census, the 2020 census remains the indispensable projection baseline. As a benchmarking check, the registered-population age structure in the latest Shanghai Statistical Yearbook (14) reproduces the district aging rates in Section 3.1.1 exactly (Fengxian 36.9%, Jiading 35.0%, Qingpu 34.5%, Pudong 33.4%, Songjiang 31.1%), and its updated figures confirm that the aging trajectory is continuing in four of the five districts (Fengxian 37.8%, Qingpu 35.0%, Pudong 33.5%, Songjiang 31.4%; Jiading stable at 34.7%). Yearbook resident-population totals (14) quantify the gap between the registered baseline and the actual service population, and net migration was incorporated explicitly (Section 2.4).

### 2.3 Assessment of equity in resource allocation

Using subdistricts or districts as units of analysis, a population-resource matrix was constructed and evaluated using three indicators. The Gini coefficient measures the degree of equality in the distribution of resources by population or area (18); *G* ≤ 0.3 indicates relative equity, and G>0.4 marks the equity warning threshold. The Theil index is decomposable and is used to identify the main sources of inequity. Emergency response time (ERT) was used as an indicator of service output equity, and the interregional Gini coefficient of ERT was calculated.

### 2.4 Population projection and resource demand estimation

The population was projected using the cohort-component method with PADIS-INT software, taking age- and sex-specific data from the 2020 Seventh National Population Census as the baseline. The study by Chen et al. (23) on multi-scenario population projections for China provided the methodological basis for the multi-scenario specification; the analysis by Vollset et al. (24) of population projections under alternative fertility and migration scenarios supported the low/medium/high multi-scenario comparative design adopted herein. International research has widely incorporated population change into forecasting of prehospital emergency demand; 'for example, a statewide projection of EMS demand in Florida through 2035 (25) demonstrated that population aging is the core driver of the growth in emergency demand. Three fertility scenarios were set: a low scenario (total fertility rate remains at the 2020 baseline 0.78–0.95), a medium scenario (total fertility rate increases linearly to reach 1.0 by 2030), and a high scenario (total fertility rate reaches 1.2 by 2030). Migration assumptions were calibrated to the actual net in-migration rates (3.5%–12.8%) of each district. The main text reports the projection results for the medium scenario. Because the census baseline refers to the household-registered population, whereas yearbook data show registered residents accounting for only 39–59% of the resident population in the five districts (Songjiang 39.3%, Jiading 41.2%, Qingpu 42.8%, Fengxian 50.7%, Pudong 58.7%) (14), they were also calibrated to each district's observed total population growth; registered-population migration statistics show net in-migration in all five districts in 2023 and 2024 (14). The low/medium/high scenarios bracket the demand uncertainty arising from this registered-vs.-resident gap.

Resource needs were estimated using a personnel-to-population ratio method based on two policy standards. Standard R_1_ requires 1 ambulance per 30,000 population (Shanghai requirement), and standard R_2_ requires 1 ambulance per 20,000 population (central urban benchmark). Total demand = forecast population × standard ratio, with staffing of 1 physician plus 3 auxiliary personnel per ambulance.

### 2.5 Spatial coverage assessment

Spatial coverage assessment was performed using QGIS 3.34 and the ORS tool. Isochrones of 5, 8, and 10 min were generated around 20 substations to identify service gaps. Because ORS isochrones are computed from average road-network speeds, they represent average traffic conditions and do not capture peak-hour congestion; the coverage results should therefore be interpreted as optimistic (best-case) estimates (see Sections 3.4.1 and 4.4).

### 2.6 Analysis of emergency medical service operational characteristics

Emergency medical service operational characteristics were analyzed for the three districts with comparable call-record fields: Qingpu (27,462 calls), Songjiang (42,879 calls), and Fengxian (9,863 calls). Disease categories, age distributions, and call time-period distributions were summarized. Although de-identified 2024 call records were obtained from all five districts, field coding differs across dispatch centers; only the three districts above provided directly comparable age and disease fields, so the findings in Sections 3.1.2–3.1.3 are presented explicitly as three-district evidence (see Section 4.4).

## 3 Results

To address the association between aging and spatial equity, this section follows a logic chain of demand—supply—gap—risk. First, the actual impact of aging on EMS resource utilization and demand structure is analyzed from the demand side (3.1); second, the spatial equity of current EMS resource allocation is assessed from the supply side (3.2); third, aging projections are combined with resource allocation standards to quantify the supply-demand gap by 2035 (3.3); and finally, the superimposed risk of aging hotspots and spatial coverage gaps is identified at the subdistrict level (3.4).

### 3.1 Aging-driven pressure on emergency care demand

#### 3.1.1 Regional differences in population aging

Based on household-registered population age structure data from 2005–2023 (Figure 1), the degree and pace of aging showed significant inter-district variation across the five districts. In 2023, the proportion of the population aged 60 years and older ranked from high to low as follows: Fengxian (36.9%), Jiading (35.0%), Qingpu (34.5%), Pudong (33.4%), and Songjiang (31.1%); relative to 2005, the increases were 19.0, 14.6, 15.9, 15.0, and 12.8 percentage points, respectively, with Fengxian rising the fastest and Songjiang the slowest. Over the same period, the proportion of the population aged 0–17 years was highest in Songjiang (15.2%) and lowest in Fengxian (10.7%), suggesting spatial heterogeneity in child-rearing potential. Using the range of proportions across four age groups as an indicator of age structure balance, Pudong (22.5 percentage points), Jiading (22.6), and Songjiang (22.8) were relatively balanced, while Qingpu (26.0) and Fengxian (28.0) showed more pronounced divergence. Given that Pudong has the largest total population among the five districts, its absolute number of older adults is also the largest. In summary, aging is spatially heterogeneous: Fengxian and Qingpu face greater relative demand pressure, whereas Pudong, despite having a mid-range aging rate, has the largest absolute older population. Throughout this study, older adults are defined as persons aged ≥60 years, following the Chinese statistical convention (65 years and above where specified); because the call records are coded in 5-year bands, the single-year count of patients aged exactly 60 years was obtained from the district dispatch centers to align the patient-side indicator with the population-side ≥60 threshold. District-level statistics for Nanhui New City are published under Pudong New Area (the former Nanhui District was merged into Pudong in 2009), so “Pudong” in this section includes Nanhui New City.

**Figure 1:**
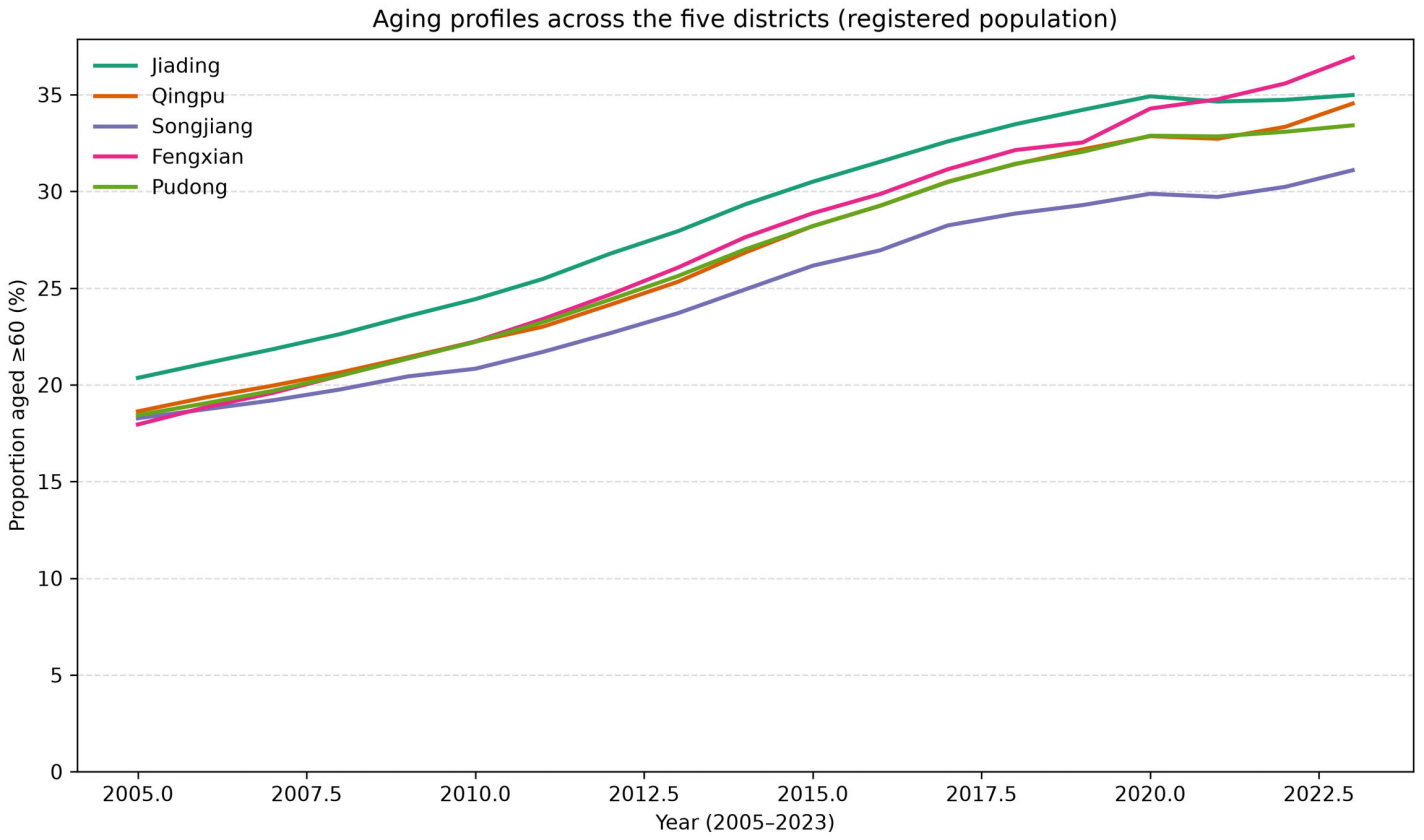
Inter-district trajectories of the proportion aged ≥60 years, 2005–2023. District-level statistics for Nanhui New City are published under Pudong New Area, into which the former Nanhui District was merged in 2009.

#### 3.1.2 Age structure of EMS patients

According to 2024 emergency call records, the age distribution of EMS patients in the three districts with comparable field coding (Qingpu, Songjiang, and Fengxian) showed a consistent pattern: older adults aged 60 years and above were the predominant users, accounting for 57.61% in Qingpu, 48.49% in Fengxian, and 47.75% in Songjiang. The middle-aged group (41–59 years) followed, at 19.85%, 24.05%, and 20.61%, respectively. Combined, the middle-aged and older population (aged 41 years and above) accounted for 77.46%, 72.54%, and 68.36% of each district, indicating that EMS resources are primarily used by the middle-aged and older population (Table 1), and that districts with higher aging rates experience greater actual operational pressure on the emergency system (Records from all five districts were obtained, but only the three districts above have directly comparable fields; the ≥60 aggregate combines the 61–65-and-older bands with the dispatch-center count of patients aged exactly 60 years; definitions follow Section 3.1.1.).

**Table 1:** Age structure of EMS patients in Qingpu, Fengxian, and Songjiang.

Age group (years)	Qingpu *n*	Qingpu %	Fengxian *n*	Fengxian %	Songjiang *n*	Songjiang %
0–5	479	1.74	214	2.17	332	1.70
6–10	230	0.84	113	1.15	206	1.05
11–15	236	0.86	106	1.07	234	1.20
16–20	432	1.57	229	2.32	675	3.45
21–25	687	2.50	362	3.67	976	4.99
26–30	1,253	4.56	495	5.02	1,083	5.54
31–35	1,471	5.36	576	5.84	1,360	6.95
36–40	1,401	5.10	613	6.22	1,323	6.76
41–45	1,267	4.61	517	5.24	1,014	5.18
46–50	1,306	4.76	561	5.69	984	5.03
51–55	1,540	5.61	718	7.28	1,084	5.54
56–60	1,785	6.50	762	7.73	1,287	6.58
61–65	1,765	6.43	820	8.31	1,204	6.16
66–70	2,546	9.27	858	8.70	1,608	8.22
71–75	2,459	8.96	794	8.05	1,471	7.52
76–80	2,428	8.84	743	7.53	1,537	7.86
81–85	2,244	8.17	582	5.90	1,276	6.52
86–90	2,292	8.35	508	5.15	1,109	5.67
91–95	1,265	4.61	224	2.27	627	3.21
96–100	336	1.22	53	0.54	152	0.78
>100	36	0.13	15	0.15	18	0.09

Percentages are within-district shares of EMS patients by age group. Because the source records code age in 5-year bands, the exact count of patients aged 60 years (within the 56–60 band) was obtained from the district dispatch centers, allowing the ≥60-year share to be computed exactly: 57.61% (Qingpu), 48.49% (Fengxian), and 47.75% (Songjiang).

#### 3.1.3 Disease spectrum of EMS patients

In the inter-district comparison of the three new towns, injury-related diseases constituted the largest category, followed by time-sensitive conditions such as circulatory and cerebrovascular diseases. This distribution suggests that the new town areas are more dependent on high-standard response times and are more sensitive to vehicle and shift allocation during morning peak hours or specific time periods. The disease spectrum structure across the three districts consistently points to a common feature: injuries and cardiovascular/cerebrovascular emergencies, both highly prevalent in the older population, are time-sensitive conditions whose treatment outcomes are highly dependent on response speed (Table 2). Therefore, aging not only quantitatively increases EMS demand but also qualitatively imposes higher requirements for response timeliness and triage accuracy.

**Table 2:** Disease categories of EMS patients in Qingpu, Fengxian, and Songjiang.

Disease category	Qingpu *n*	Qingpu %	Fengxian *n*	Fengxian %	Songjiang *n*	Songjiang %
Injury-related diseases	9,275	33.78	3,658	37.09	7,551	38.60
Other	3,706	13.50	1,393	14.12	3,536	18.08
Circulatory system	2,504	9.12	895	9.07	1,775	9.07
Respiratory system	2,418	8.81	819	8.30	1,174	6.00
Digestive system	2,185	7.96	692	7.02	1,267	6.48
Cerebrovascular diseases	1,472	5.36	563	5.71	792	4.05
Physical/chemical factor–related diseases	1,242	4.52	381	3.86	671	3.43
Genitourinary system diseases	977	3.56	392	3.97	498	2.55
Neoplasms	783	2.85	209	2.12	516	2.64
Obstetric and gynecological diseases	722	2.63	141	1.43	305	1.56
Other nervous system diseases	461	1.68	168	1.70	629	3.22
Endocrine and metabolic diseases	439	1.60	134	1.36	272	1.39
ENT and ophthalmic diseases	315	1.15	55	0.56	86	0.44
Pediatric diseases	311	1.13	112	1.14	201	1.03
Mental and behavioral disorders	276	1.01	65	0.66	136	0.70
Hematological diseases	165	0.60	44	0.45	47	0.24
Connective tissue, rheumatic, and skin diseases	133	0.48	39	0.40	83	0.42
Infectious diseases	70	0.25	7	0.07	16	0.08
Refusal to provide medical history	4	0.01	2	0.02	5	0.03
Multiple diagnoses	—	—	94	0.94	—	—

Percentages are within-district shares. “Multiple diagnoses” aggregates Fengxian records coded with two or more disease categories (*n* = 94; 0.94%). Qingpu and Songjiang source tables list single-category codes only. Em dash (—) indicates not reported in the source table.

### 3.2 Current spatial inequity in emergency resource allocation

#### 3.2.1 Resource inventory

As of the end of 2024, Shanghai had a total of 210 EMS substations, with 102 located across the five districts, of which 20 were in the new town core areas (Figure 2). Compared with the central urban area, substation density in the new towns was significantly lower, with distribution concentrated in traditional town centers, while peripheral areas and regions fragmented by major transportation corridors or waterways remained service blind spots. The five districts had a total of 598 ambulances. The 20 new town EMS substations were concentrated in the historical town centers, with density markedly lower than that of the central urban area.

**Figure 2:**
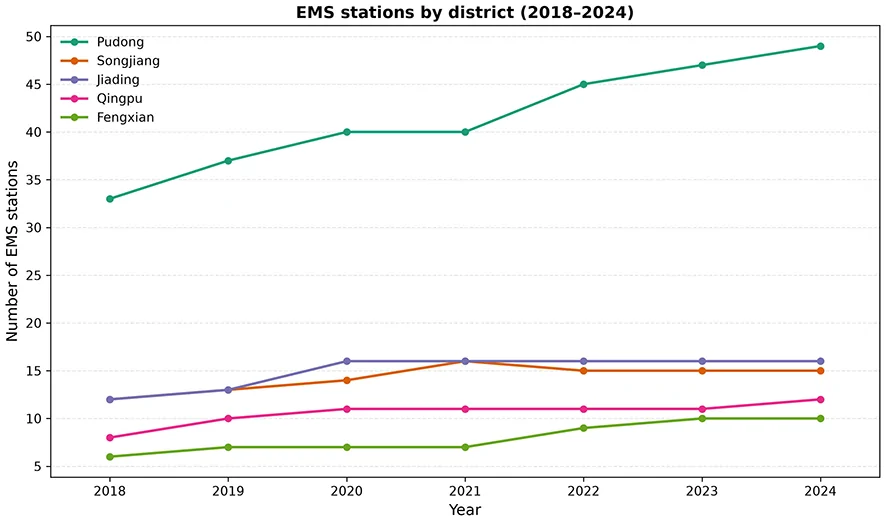
Distribution of EMS substations.

#### 3.2.2 Gini coefficient

On a per capita basis, the Gini coefficients for all three resource types were below 0.3 (physicians: 0.10, ambulances: 0.09, substations: 0.03). However, when all 16 Shanghai districts were included, the area-based Gini coefficient for substations reached 0.61 (Table 3), reflecting the gradient in substation density between the central urban area and the new towns. When calculated for the five new town districts only, the area-based Gini coefficient for substations was 0.21, indicating a moderate degree of intra-district spatial inequity.

**Table 3:** Gini coefficients of EMS resources, 2024.

Item	Physicians	Ambulances	Stations
By population (5 districts only)	0.10	0.09	0.03
By population (incl. all 16 districts)	0.05	0.04	0.01
By area (5 districts only)	0.13	0.24	0.21
By area (incl. all 16 districts)	0.08	0.11	0.61

Gini coefficients range from 0 (perfect equality) to 1 (perfect inequality). Values are reported for physicians, ambulances, and stations, allocated by population or by area, for the five new-city districts only and with all 16 Shanghai districts included.

#### 3.2.3 Theil index

The Theil index decomposes inequality into between-district and within-district components (Table 4). For all three resource types, within-district inequality was the primary contributor (ambulances: 0.25, substations: 0.21, physicians: 0.12), while the east-west regional difference was negligible (0.00–0.02). This indicates that the main equity challenge lies in the intra-district distribution of substations rather than inter-district resource allocation. In other words, the key to equity is not which district, but which subdistrict within a district.

**Table 4:** Theil index of EMS resources by district, 2024.

Region	Ambulances	Stations	Physicians
Between-district inequality	0.25 (Relatively equitable)	0.21 (Relatively equitable)	0.12 (Relatively equitable)
East-West regional difference	0.02 (Relatively equitable)	0.01 (Relatively equitable)	0.00 (Absolutely equitable)

Evaluation criteria—*T* = 0, Absolutely equitable; 0 <*T* < 0.3, Relatively equitable; 0.3 ≤ *T* < 1, Moderately inequitable; *T* ≥ 1, Highly inequitable. All between-district and regional inequality values fall below 0.3, indicating relatively equitable resource allocation across districts. The near-zero east-west regional differences (0.00–0.02) suggest negligible geographic disparity at the macro scale.

#### 3.2.4 Service output equity: emergency response time

ERTs in all five districts were below 720 s (12 min), with interval Gini coefficients 0.02–0.03. Mean values by district: Songjiang 535 s, Pudong 629 s, Jiading 637 s, Fengxian 649 s, Qingpu 661 s (Table 5). Stratified analyses revealed significant temporal variation: the 4:00–7:00 early-morning period had the longest ERT (median 663 s, 14.0%-15.6% slower than 20:00–23:00, *p* < 0.001), and Monday was the slowest among weekdays (4.1% slower than Sunday, *p* < 0.001). In two of the three districts, ERT did not differ significantly between cardiac arrest and non-urgent patients (*p* > 0.05), suggesting that the dispatch system may have failed to consistently prioritize time-sensitive cases. This further indicates that the efficiency of the current triage dispatch system has not adequately reflected the differentiated needs of different patient groups in the context of population aging.

**Table 5:** EMS response efficiency by region, 2024.

Region	Permanent resident population (10,000 persons)	Emergency response time (s)
Fengxian	114.43	649
Qingpu	127.88	661
Jiading	188.61	637
Songjiang	197.35	535
Pudong	581.11	629
Citywide (Shanghai)	2,487.45	598

Population is permanent resident population in units of 10,000 persons. Emergency response time is reported in seconds. Pudong New Area includes Nanhui New City (the former Nanhui District was merged into Pudong in 2009).

### 3.3 Resource demand gap driven by population aging

#### 3.3.1 Population aging will continue to rise

All three fertility scenarios (low, medium, and high) show a structural upward trend in the aging rate across the five new towns. Taking the Jiading New Town core area as an example, the low/high scenarios produce a variation range of approximately ±10% by 2035 (Table 6).

**Table 6:** Number and share of the older population (aged ≥65), 2020–2035.

Year	Low scenario		Medium scenario		High scenario	
	Population aged ≥65	Share of total population (%)	Population aged ≥65	Share of total population (%)	Population aged ≥65	Share of total population (%)
2020	81,956	13.18	81,956	13.18	81,956	13.18
2021	88,547	14.00	88,682	13.93	88,811	13.86
2022	94,872	14.75	95,150	14.60	95,420	14.46
2023	100,957	15.45	101,391	15.21	101,818	14.97
2024	106,838	16.10	107,441	15.75	108,030	15.43
2025	112,536	16.70	113,321	16.25	114,098	15.82
2026	118,155	17.28	119,132	16.72	120,109	16.18
2027	123,488	17.82	124,677	17.13	125,870	16.47
2028	128,583	18.31	130,000	17.49	131,430	16.71
2029	133,474	18.76	135,135	17.81	136,821	16.90
2030	138,192	19.19	140,113	18.10	142,082	17.06
2031	142,968	19.62	145,174	18.38	147,448	17.20
2032	147,478	20.00	149,990	18.62	152,593	17.31
2033	151,773	20.36	154,609	18.82	157,575	17.38
2034	155,890	20.69	159,084	19.00	162,447	17.42
2035	159,869	20.99	163,446	19.15	167,243	17.44

Figures are projections for the core area of Jiading New City under the low, medium, and high scenarios. Population aged ≥65 is reported as person counts; shares are percentages of the total population.

#### 3.3.2 Jiading case: a generalizable aging trajectory

The population aged 65 years and older in the Jiading New Town core area shows a continuous growth trend from 2020 to 2035. Under the medium scenario (baseline scenario, assuming total fertility rate increases linearly to 1.0 by 2030), the proportion of the older population increases from 13.18% in 2020 to 19.15% in 2035; under the low scenario (maintaining the 2020 baseline fertility rate of 0.78–0.95), it would reach 20.99%; and under the high scenario (total fertility rate reaching 1.2 by 2030), it would reach 17.44%. All three scenarios indicate a sustained upward trend in the aging rate. Based on the medium-scenario population projection and R_1_/R_2_ standards, the demand for ambulances in each new town increases by 2035. Nanhui increases from 18 to 24 vehicles, and Songjiang from 28 to 35 vehicles (Table 7).

**Table 7:** Forecasted EMS resource demand in the five new cities, 2035.

New city	Projected population (2035)	Standard R1		Standard R_2_	
		Ambulances	Personnel	Ambulances	Personnel
Jiading	853,609	29	29+87	43	43+129
Qingpu	454,290	16	16+48	23	23+69
Songjiang	1,030,825	35	35+105	52	52+156
Fengxian	414,904	14	14+42	21	21+63
Nanhui	704,923	24	24+72	36	36+108

R1 and R_2_ denote two EMS resource-allocation standards. Personnel is reported as “physicians + supporting staff” (e.g., 29+87).

#### 3.3.3 Resource gap under policy standards

Based on population scenarios (low/medium/high) from the PADIS-INT cohort-component method and two types of policy standards (R_1_: 1 ambulance per 30,000 population; R_2_: 1 ambulance per 20,000 population), ambulance and personnel requirements by 2035 were estimated at the district level. The medium scenario served as the baseline, while the low and high scenarios were used to establish a reasonable range (Table 8). Taking Jiading as an example, the current R1 standard requires 24 vehicles in 2025 and 29 vehicles in 2035, yielding a ten-year gap of 5 vehicles; under the R_2_ standard, demand increases from 35 to 43 vehicles, yielding a gap of 8 vehicles.

**Table 8:** Forecasted pre-hospital EMS resource demand in the core areas of the five new cities.

New city	Year	Projected population	Based on standard I (R1 = 1/30,000)		Based on standard II (R_2_ = 1/20,000)	
			Ambulances (vehicles)	Personnel (physicians + support)	Ambulances (vehicles)	Personnel (physicians + support)
Jiading New City	2025	697,281	24	24+72	35	35+105
	2030	774,292	26	26+78	39	39+117
	2035	853,609	29	29+87	43	43+129
Qingpu New City	2025	406,972	14	14+42	21	21+63
	2030	431,916	15	15+45	22	22+66
	2035	454,290	16	16+48	23	23+69
Songjiang New City	2025	827,237	28	28+84	42	42+126
	2030	926,724	31	31+93	47	47+141
	2035	1,030,825	35	35+105	52	52+156
Fengxian New City	2025	396,862	14	14+42	20	20+60
	2030	407,837	14	14+42	21	21+63
	2035	414,904	14	14+42	21	21+63
Nanhui New City	2025	516,753	18	18+54	26	26+78
	2030	605,808	21	21+63	31	31+93
	2035	704,923	24	24+72	36	36+108

R1 = 1 ambulance (and corresponding staffing) per 30,000 population; R_2_ = 1 per 20,000. Personnel is reported as “physicians + supporting staff” (e.g., 24+72).

#### 3.3.4 Scenario analysis and elastic redundancy

The above gaps are based on the medium scenario (TFR reaching 1.0 by 2030, with migration rates set according to each district's actual population growth rate of 1.9%−12.8%). Sensitivity analysis indicates that: (1) a decline in fertility to the low scenario (TFR maintained at 0.78–0.95) would raise the aging rate (e.g., Jiading reaching 20.99%, 1.84 percentage points higher than the medium scenario), but the total population would also be smaller under the low scenario (761,627 vs. 853,609 in Jiading), so the impact on demand requires comprehensive assessment; (2) if in-migration exceeds expectations (high scenario), the expansion of the total population would directly increase absolute demand. It is recommended that elastic redundancy be reserved in total allocation to accommodate peak demand arising from the combined effects of low fertility and high migration.

### 3.4 Superimposed risk of aging hotspots and spatial coverage gaps

The preceding three sections have examined the association between aging and the spatial equity of EMS resources from the demand side, supply side, and gap side, respectively. This section further refines the analytical scale from the district level to the subdistrict level, directly testing whether aging hotspots and spatial coverage blind spots overlap within the same spatial units.

#### 3.4.1 Coverage overview and blind spots of each new town

QGIS isochrone analysis showed that the 8 min coverage of the 20 EMS substations across the five new towns covered approximately 60%−75% of the core built-up areas. Blind spots were concentrated in the northern industrial area of Jiading, the western waterway network area of Qingpu, the eastern industrial area and Sheshan tourist area of Songjiang, the northeastern residential area of Fengxian, and the hinterland areas between substations in Nanhui. Specifically, even the 10 min coverage of Jiading did not reach the northernmost area; Qingpu, fragmented by waterways, showed irregular patchy coverage; Songjiang's old town and university town areas had the highest coverage efficiency, but the eastern area and Sheshan were relatively underserved; the northeastern area of Fengxian overlapped considerably with future population influx zones; and there was a pronounced “service gap” between the core area and functional zones of Nanhui. As these isochrones assume average traffic conditions (Section 2.5), real-world coverage contracts further during morning and evening peaks, and the blind spots identified here are lower-bound estimates of the true spatial inequity. As a sensitivity check, published congestion assessments report peak-period travel times in Shanghai to be approximately 50% longer than free-flow conditions (Amap Annual Traffic Report 2024 (39); TomTom Traffic Index 2024 (40)); applying this slowdown implies that the 8 min service area contracts during rush hours to roughly 5.3 min of reach (about the present 5 min coverage) and the 10 min area to roughly 6.7 min. This order-of-magnitude estimate is consistent with the observed 4:00–7:00 ERT lengthening of 14.0%−15.6% vs. the 20:00–23:00 baseline (Section 3.2.4).

#### 3.4.2 Spatial overlap of aging hotspots and coverage gaps

Overlaying the 8 min coverage threshold with subdistrict-level aging rates (aged ≥65 years) revealed overlap patterns across all five new towns. The aging rate in Shuyuan Town, Pudong, reached 23%, with its southern area falling within the 8 min coverage blind spots of the Shengang and Nicheng substations. The aging rate in Zhenxin Subdistrict, Jiading, reached 20.5%, located at the boundary between the service areas of the Jiading urban substation and the Malu North substation. The Sheshan tourist area in Songjiang had an aging rate exceeding 18%, with insufficient 8 min coverage. Xianghuaqiao Subdistrict in Qingpu and the northeastern residential area of Fengxian similarly exhibited a pattern of “high aging rate × low coverage” (Figure 3). This spatial overlap provides the most direct micro-level evidence that aging exacerbates spatial inequity: at the subdistrict scale, the older adult population most in need of emergency care is precisely located where EMS resources are least able to reach quickly. To make the spatial dimension of equity directly visible, the station distribution is mapped against the new town boundaries in Figure 2, the 8- and 10 min isochrones are mapped in Figure 3, and a composite overlay map for all five new towns (Supplementary Figure S1) juxtaposes 8 min isochrone coverage and substation locations with district-level aging rates, allowing the aging–coverage inequity to be read directly from the map.

**Figure 3:**
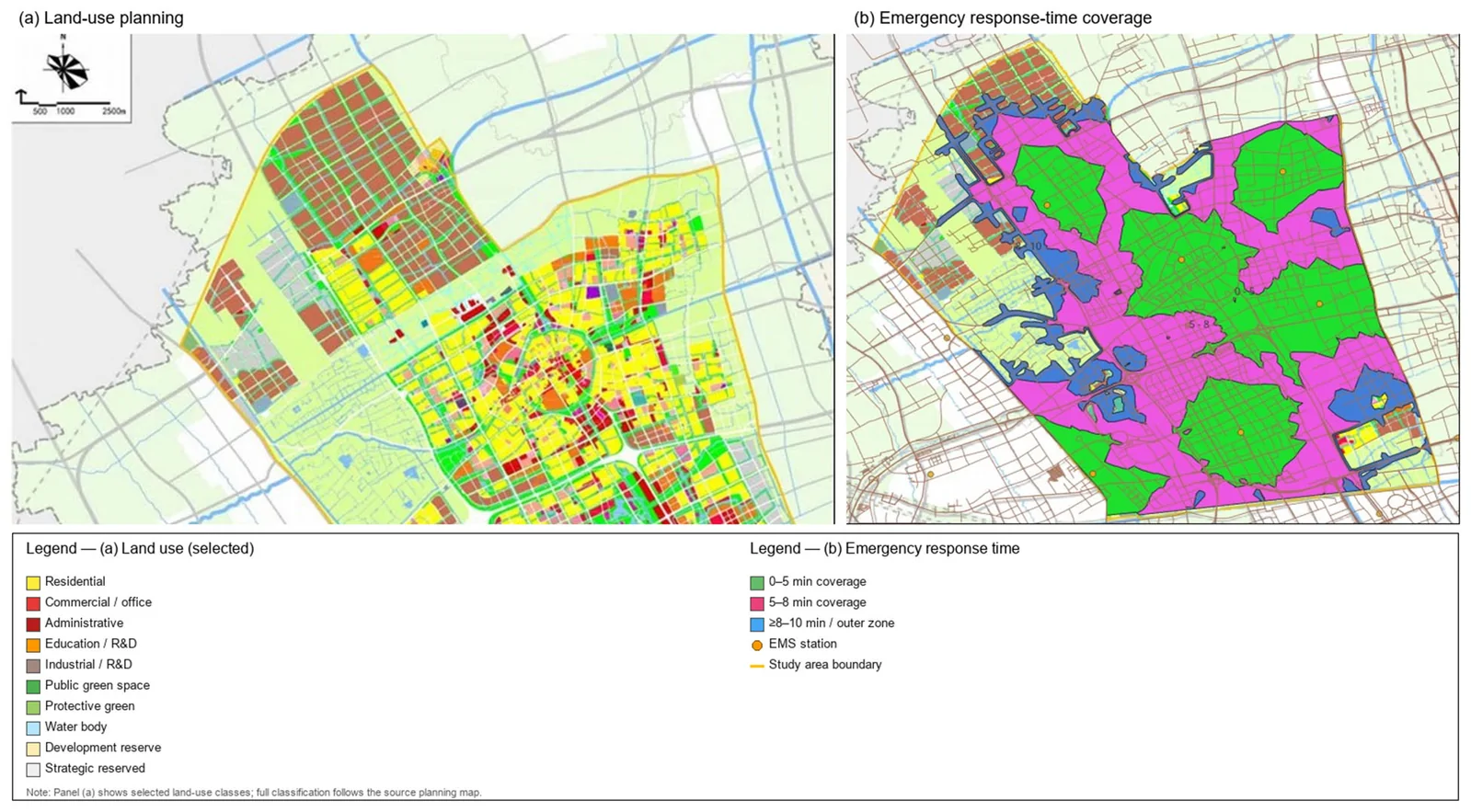
Coverage areas of EMS substations in Jiading New Town.

## 4 Discussion

This study identified a dual-track pattern: equity in the population dimension but inequity in the spatial dimension. The following discussion analyzes how aging interacts with this pattern through three mechanisms.

### 4.1 Does aging exacerbate the spatial inequity of EMS resources?

The five new towns exhibit a dual-track characteristic of being “relatively equitable in the population dimension but insufficiently equitable in the spatial dimension.” Aging superimposes on this inequity through three pathways: first, an increase in total demand (older adults are the primary user group); second, a rising proportion of time-sensitive disease categories that are more responsive-time-dependent; and third, spatial overlap of aging hotspots and coverage gaps amplifying localized accessibility disparities. Aging exacerbates spatial inequity primarily through three mechanisms. From the population dimension, aging does not significantly worsen equity; however, from the spatial and future dimensions, the three mechanisms progressively emerge.

Mechanism 1: Dual elevation of demand volume and demand structure. Beyond chronological age, frailty—a syndrome of multisystem physiological reserve decline—has become a stronger predictor of adverse outcomes than age itself (26). The population aged 65 years and older in Jiading is projected to reach 19.15% by 2035, with ambulance demand under the R_1_ standard increasing to 29 vehicles. However, the burden of frailty in this aging population may exceed age-based estimates, implying that actual resource requirements may be higher than our projections.

Mechanism 2: Shift in the disease spectrum toward time-sensitive conditions. In Fengxian, injuries account for 37.09%, circulatory system diseases for 9.07%, and cerebrovascular diseases for 5.71%. This pattern is consistent with the broader challenge of geriatric trauma: older adults frequently sustain severe injuries even from low-energy mechanisms such as falls, which are underestimated by standard prehospital assessment tools (27). Physiological changes—blunted heart rate response to hypovolemia and sarcopenia masking injury signs—mean that injury severity in older patients is often underestimated at the dispatch stage (27). Socioeconomic disparities further amplify this risk: low-income older adults face higher fall risk due to inadequate home safety modifications, malnutrition, and social isolation (15). Similarly, social determinants of health also influence cognitive rehabilitation outcomes in older adults with traumatic brain injury (28).

Mechanism 3: Spatial overlap of aging subdistricts and coverage gaps. In subdistricts with aging rates exceeding 18% (e.g., Shuyuan 23%, Zhenxin 20.5%), population-based equity masks deficiencies in spatial coverage. The area-based Gini coefficient for substations within the five districts is 0.21, indicating that intra-district substation distribution is the primary spatial challenge.

### 4.2 Implications for international aging and emergency care research

The parallel study by Li et al. (29) on spatial equity of primary healthcare resources in Shanghai's new towns provides directly comparable evidence within the same city, and its findings are consistent with our results in terms of spatial patterns. Furthermore, international research has documented ‘ambulance deserts'—areas where EMS substations cannot reach population centers within 25 min—affecting approximately 4.5 million people in the United States alone, with children and socially vulnerable populations disproportionately impacted (30). The maximum coverage models of Grot (31) and Hashtarkhani et al. (32) indicate that emergency resources must be dynamically adjusted according to population distribution (33, 34). Our finding—that the area-based Gini coefficient for substations reaches 0.61 when all 16 Shanghai districts are included—is consistent with these international patterns of urban-rural disparity. International research emphasizes that EMS site selection should transition from single-efficiency optimization to multi-constraint, multi-objective optimization (35, 36).

This study has direct relevance for rapidly urbanizing countries in the Global South, including India, Indonesia, and Brazil. Importantly, the methodological toolkit employed in this study was designed to be replicable: PADIS-INT is a free population projection platform, QGIS and ORS tools provide open-source spatial coverage analysis, and the Gini coefficient and Theil index are widely recognized universal equity indicators. This enables health authorities in resource-constrained regions to conduct similar baseline assessments without expensive software. The clinical effectiveness of telemedicine in trauma care has been validated in systematic reviews, offering a feasible pathway for prehospital triage support for older patients in resource-limited settings (37).

### 4.3 Policy recommendations and implementation pathways

(1) Substation layout optimization: prioritize the addition of substations or rapid response units in subdistricts with insufficient 5–8 min coverage overlapping with aging hotspots. (2) Vehicles and shifts: deploy flexible vehicle teams during the early morning peak (4:00–7:00) and on Mondays to reduce ERT during high-risk periods. (3) Tiered R standards: implement R_2_ configuration in new town core areas and R_1_ in peripheral areas, with an annual dynamic calibration mechanism. (4) Vulnerability prioritization: incorporate frailty and SDoH screening results into dispatch priority, and pilot tele-triage support for older patients. At the same time, attention should be paid to the transportation service needs of older adults with cognitive impairment in long-term care facilities (38).

### 4.4 Limitations

This study has several limitations. First, the Gini coefficient was calculated using administrative districts as units, which do not correspond exactly to the core boundaries of the five new towns. Second, the population projections were based on the 2020 Seventh National Census household-registered population, which may underestimate the population that migrated into the new towns after 2020. Third, the stratified ERT analysis covered only three districts. Fourth, traffic conditions were based on historical averages and did not account for peak-hour congestion. Fifth, there is a potential ecological fallacy. Sixth, a cost-benefit analysis was not conducted. Seventh, this study did not include individual-level social determinants of health data such as socioeconomic status, living conditions, or social support networks, factors that have been demonstrated to significantly influence injury risk, EMS utilization patterns, and post-discharge outcomes in older adults. Eighth, transportation barriers faced by vulnerable populations—such as older adults in long-term care facilities—were not explicitly modeled. In addition, published statistics do not report the new-town-core areas separately from their host districts, so the district-level equity indicators should be read as upper-scale approximations of new-town conditions rather than exact measures.

### 4.5 Future research directions

Dynamic resource allocation and real-time dispatch models, frailty-informed demand forecasting, intelligent EMS assessment incorporating SDoH indicators, and the extension of the dual-track framework to other rapidly urbanizing new towns are important future research directions. Telemedicine-based prehospital triage support, particularly for underserved older populations, warrants further investigation. Methodologically, future work should incorporate time-dependent isochrones that model peak-hour congestion (33, 34), extend the call-record analysis to the remaining two districts as data become available, and validate the registered-population projections against intercensal resident-population estimates.

## 5 Conclusion

Based on the current EMS operational status in 2024 and population projections from 2020 to 2035, this study systematically assessed the spatial equity of resource allocation and its association with population aging.

First, the dual-track pattern reveals: population-dimension allocation is equitable (physician Gini = 0.10, substation Gini = 0.03), but area-based substation allocation shows moderate inequity within the five new towns (Gini = 0.21), expanding to 0.61 when all 16 districts are included. Second, aging-driven demand growth is significant: the population aged ≥65 in Jiading increases from 13.18% to 19.15% (2035), and ambulance demand under the R1 standard in Jiading New Town is projected to increase from 24 vehicles in 2025 to 29 vehicles in 2035. Third, spatial overlap risk: highly aged subdistricts overlap with 8 min coverage gaps. Fourth, dispatch-level inequity: ERT did not differ significantly by clinical urgency in two of the three districts (*p* > 0.05), suggesting that the current triage dispatch system may not adequately prioritize time-sensitive cases.

These findings reveal a structural contradiction in resource allocation within megacity new town areas: aging-driven demand growth has outpaced the spatial expansion of emergency infrastructure. The dual-track equity framework developed in this study can be extended to other rapidly urbanizing regions. Integrating frailty assessment, SDoH indicators, and geriatric-specific triage protocols into prehospital emergency care is a priority pathway for improving equity and clinical outcomes.

## Data Availability

The original contributions presented in the study are included in the article/Supplementary material, further inquiries can be directed to the corresponding author.
